# Predicting 30-Day Mortality Using ST-Segment Elevation Resolution in ST-Elevation Myocardial Infarction Patients Undergoing Primary Percutaneous Coronary Intervention: An Indian Scenario

**DOI:** 10.7759/cureus.38663

**Published:** 2023-05-07

**Authors:** Shankar Chandra Mandal, Bhushan Shah, Lokendra Rekwal, Vishal Batra

**Affiliations:** 1 Department of Cardiology, Institute of Post Graduate Medical Education and Research, Kolkata, IND; 2 Department of Cardiology, Mahatma Gandhi Memorial (MGM) Medical College, Indore, IND; 3 Department of Cardiology, Govind Ballabh (GB) Pant Hospital, New Delhi, IND

**Keywords:** stemi, prognosis, percutaneous coronary intervention, mortality, electrocardiography

## Abstract

Background: The relationship between ST-segment elevation (STE) resolution and 30-day mortality has been evaluated, although limitedly, in non-Indian patients undergoing primary percutaneous coronary intervention (pPCI). We aimed to evaluate the prognostic utility of STE resolution in predicting 30-day mortality in Indian patients undergoing pPCI for ST-elevation myocardial infarction (STEMI).

Materials and methods: This prospective, single-center, observational study investigated the correlation between 30-day mortality rate and extent of STE resolution in real-world Indian patients undergoing pPCI for STEMI. A total of 64 patients underwent pPCI for STEMI at a tertiary care center in India. The patients were classified into three groups based on the extent of STE resolution: complete resolution (≥70%), partial resolution (30-70%), and no resolution (<30%). The primary endpoint of the study was occurrence of major adverse cardiovascular events consisting of all-cause death, reinfarction, disabling stroke, and ischemia-induced target vessel revascularization at 30 days follow-up.

Results: The study enrolled 56 patients. The mean age of patients was 59.7±6.8 years and there were 46 (82.1%) males. Complete STE resolution (≥70%) occurred in 7.1%, partial resolution (<70-30%) in 82.1% and no resolution (<30%) in 10.7%. The mortality rate was 2.1% and 33.3% in patients with partial and no STE resolution. No mortality was seen in patients with complete STE resolution. The 30-day survival analysis revealed significant differences between the three groups (P<0.01). STE resolution served as an independent predictor of 30-day mortality across all clinical variables, including patients with post-PCI thrombolysis in myocardial infarction (TIMI) 3 flow.

Conclusions: Persistent STE after PCI is a reliable indicator of 30-day mortality in real-world STEMI patients. The extent of STE resolution can be used as a simple and affordable tool to stratify patients by the risk of mortality soon after the acute event. Due to their higher mortality at 30 days follow-up, individuals with persistent STE should be the focus for further treatment interventions.

## Introduction

ST-elevation myocardial infarction (STEMI) is the result of transmural myocardial ischemia which, in most cases, occurs due to occlusion of an epicardial coronary artery [1]. The preferred reperfusion technique for patients with STEMI is primary percutaneous coronary intervention (pPCI) [2]. However, with the increasing use of stents for complex lesions, studies have shown that the initial success of angioplasty does not ensure long-term angiographic patency [3,4]. Although most patients experience positive clinical outcomes following successful pPCI, 2.7-8% are at risk of 30-day mortality [5].

Over the past few years, several prognostic models and risk scores including the thrombolysis in myocardial infarction (TIMI) score [6], the Zwolle score [7], the Evaluation of Methods of Management of Acute Coronary Events (EMMACE) score [8], the Global Registry of Acute Cardiac Events (GRACE) score [9], and machine-learning based models [10] have been developed to predict 30-day mortality following STEMI. However, they entail certain limitations [10,11]. The validity of 12-lead electrocardiogram (ECG), a readily available instrument, is already proven to identify STEMI patients at high risk for adverse outcome after pPCI. ST-segment changes indicate epicardial flow and can therefore convey data beyond angiography alone [12].

The correlation between the extent of ST-segment elevation (STE) resolution and subsequent mortality has been established [13]. Incomplete STE resolution may occur in up to one-third of patients undergoing pPCI, regardless of TIMI 3 flow restoration [14]. The superiority of STE resolution over TIMI score in predicting the outcome of a successful pPCI has been established in literature [15]. Furthermore, evaluating the extent of STE resolution does not involve significant patient risk, cost, or specialized personnel. Therefore, investigated whether early STE resolution, assessed at the end of pPCI, has prognostic value in STEMI population.

## Materials and methods

Study design and population

This prospective, single-center, observational study was carried out at a tertiary care center in India. A total of 64 patients underwent pPCI for STEMI at our institute. The study received approval from the Institutional Ethics Committee (approval Inst/IEC/704) and was conducted in accordance with the Declaration of Helsinki. Written informed consent was obtained from all participants before enrollment. Patients who presented clinical symptoms of myocardial infarction (MI) for <24 h, ≥1 mm ST elevation in two contiguous leads or high-grade angiographic stenosis associated with regional wall motion abnormalities were included. Patients with chronic kidney disease (glomerular filtration rate <60/min), need for urgent coronary artery bypass graft, saphenous vein graft infarct lesion, left bundle-branch block although acute MI was diagnosed based on the clinical and biomarker data or confirmed by coronary angiography, previous STEMI six months ago or less, or non-STEMI were excluded from the study. The patients were classified into three groups based on the extent of STE resolution: complete resolution (≥70%), partial resolution (30-70%), and no resolution (<30%).

Study procedure and medication

Coronary angiography, pPCI and peri-procedural care were performed according to standards. Drug-eluting stents were used in most cases. Aspiration thrombectomy was performed as per individual case indication. Heparin was administered during PCI (3000 U loading dose; 70-100 U/kg bolus dose). Peri-procedural therapy involved premedication with loading doses of aspirin (325 mg); clopidogrel (600 mg), prasugrel (60 mg) or ticagrelor (180 mg); and atorvastatin (80 mg).

Data collection and follow-up

Clinical characteristics, ECG parameters, angiographic and procedural data were collected using a pre-designed, pre-tested and semi-structured schedule by the investigator. ECG was performed before and 90-120 min after the first balloon inflation. ST-segment resolution was assessed in ECGs recorded 90-120 min after the first balloon inflation. Epicardial blood flow in the infarct-related artery and myocardial perfusion grade were graded according to the TIMI group definitions. Follow-up data were collected via telephonic communication or clinical visit at 30 days by personnel unaware of the status of reperfusion or ST-segment resolution at the end of pPCI. Patients who had cardiac complaints underwent clinical, ECG, and laboratory evaluation.

Clinical endpoints and definitions

The primary endpoint was the occurrence of major adverse cardiovascular events (MACE), defined as the composite of death, myocardial re-infarction, disabling stroke, and repeat target vessel revascularization for ischemia, at 30 days follow-up. Myocardial reinfarction was defined by recurrent clinical signs and symptoms of ischemia distinct from the index event, along with concomitant ECG changes and serum biomarker evidence of myocardial necrosis [16]. Disabling stroke was defined as stroke requiring inpatient rehabilitation or skilled nursing care [17]. Target vessel revascularization was defined as unplanned repeat PCI or bypass graft implantation for stenosis in a different area of the vessel treated at the index PCI [18].

Statistical analysis

Statistical analyses were performed using Statistical Package for Social Sciences version 20.0 (IBM, Armonk, NY, USA) and Stata/SE (version 9.2). All categorical variables are depicted using relative frequency distributions. Continuous data are presented as the mean ± SD. Categorical variables were compared using chi-square statistics or Fisher’s exact test, while one-way ANOVA or Kruskal-Wallis test was used for continuous and ordinal variables, as appropriate. Event rates were determined and displayed with Kaplan-Meier methodology and compared with the log-rank test. Independent predictors of elevated ST-segment resolution and 30-day mortality were analyzed using multivariate logistic regression analysis.

## Results

**Figure 1:**
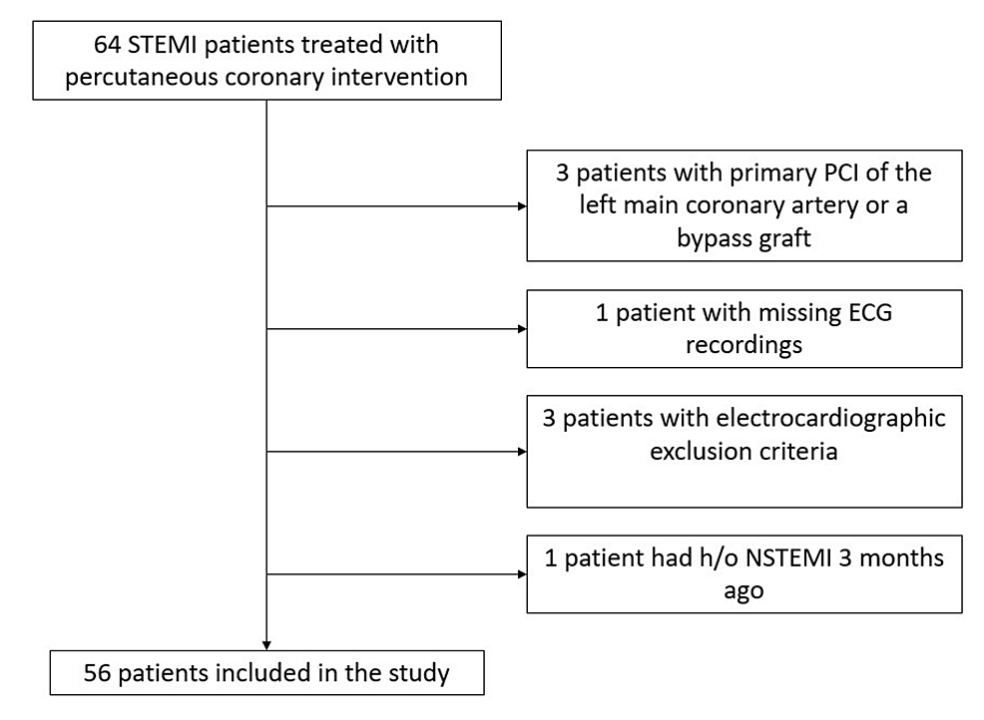
Study flow chart STEMI: ST-elevation myocardial infarction; NSTEMI: non-ST-elevation myocardial infarction; PCI: percutaneous coronary intervention; h/o: history of

The study prospectively enrolled a total of 64 STEMI patients who underwent pPCI at our institute between November 2014 and October 2015. Patient enrollment is represented in Figure 1.

Of the 56 patients included for analyses, 46 (82.1%) were male. The mean age at inclusion was 59.7±6.8 years. Of the entire cohort, six (10.7%) had a severely depressed (<35%) left ventricular ejection fraction (LVEF). Baseline characteristics and the clinical presentation are depicted in Table 1 and Table 2, respectively. 

**Table 1:** Baseline characteristics of patients BMI: body mass index; CAD: coronary artery disease

Characteristics	
Number of patients, n	56
Age (years), mean ± SD	59.7±6.8
Age distribution	
≤50 years	6 (10.7%)
51–55 years	7 (12.5%)
56–60 years	15 (26.8%)
61–65 years	21 (37.5%)
66–70 years	5 (8.9%)
>70 years	2 (3.6%)
Male, n (%)	46 (82.1%)
Female, n (%)	10 (17.9%)
BMI (kg/m^2^), mean ± SD	23.8±2.2
BMI distribution (kg/m^2^)	
18.5–23	25 (44.6%)
23–27.5	27 (48.2%)
≥27.5	4 (7.1%)
Cardiovascular risk	
Smoking, n (%)	39 (69.6%)
Diabetes mellitus, n (%)	25 (44.6%)
Hypertension, n (%)	35 (62.5%)
Dyslipidemia, n (%)	13 (23.3%)
Family history of CAD, n (%)	10 (17.9%)

**Table 2:** Clinical presentation of patients LAD: left anterior descending artery; LCX: left circumflex artery; LVEF: left ventricular ejection fraction; RCA: right coronary artery; TIMI: thrombolysis in myocardial infarction

Clinical Presentation		
Total ischemic time (mins), n (%)		
<180		8 (14.3%)
180–360		21 (57.1%)
>360		16 (28.6%)
Infarct location, n (%)		
Anterior		35 (62.5%)
Non-anterior		21 (37.5 %)
Culprit vessel, n (%)		
LAD		35 (62.5%)
RCA		17 (30.4%)
LCX		4 (7.2%)
Number of vessels, n (%)		
Single vessel		21 (37.5%)
Multiple vessel	2	21 (37.5%)
	3	14 (25.0%)
Killip class, n (%)		
Class I		34 (60.7%)
Class II		17 (30.4%)
Class III		5 (8.9%)
Lipid profile (median) (mg/dL)		
Low density lipoprotein cholesterol		115
Triglycerides		169
High density lipoprotein cholesterol		40
Total cholesterol		190
LVEF, n (%)		
<35%		6 (10.7%)
35–50%		40 (71.4%)
>50%		10 (17.9%)
Pre-TIMI flow, n (%)		
TIMI-0		20 (35.7%)
TIMI-1		30 (53.6%)
TIMI-2		6 (10.7%)
TIMI-3		0 (0.0%)
Post-TIMI flow, n (%)		
TIMI-0		1 (1.79%)
TIMI-1		2 (3.57%)
TIMI-2		12 (21.43%)
TIMI-3		41 (73.21%)

The patients were classified into three groups based on the extent of STE resolution: complete resolution (≥70%), partial resolution (30-70%), and no resolution (<30%). The patient characteristics stratified by STE resolution are given in Table 3.

**Table 3:** Patient characteristics stratified by the extent of STE resolution BMI: body mass index; LVEF: left ventricular ejection fraction; STE: ST-segment elevation; TIMI: thrombolysis in myocardial infarction

Variable	Extent of STE resolution			P value
	No Resolution (n=6)	Partial Resolution (n=46)	Complete Resolution (n=4)	
Total study population (n=56)				
Age, years, mean ± SD	60 (6.3)	59.8 (7.2)	58.5 (3)	0.840
Male/female, n (%)	5 (88.3%)	38 (82.6%)	3 (75.0%)	0.617
	1 (16.7%)	8 (17.4%)	1 (25.0%)	
Hypertension, n (%)	5 (88.3%)	29 (63.0%)	1 (25.0%)	0.006
Diabetes mellitus, n (%)	4 (66.8%)	19 (41.3%)	2 (50.0%)	0.489
Hypercholesterolemia, n (%)	3 (50.0%)	9 (19.6%)	1 (25.0%)	0.159
Smoker, n (%)	3 (50.0%)	33 (71.7%)	3 (75.0%)	0.242
Family history of cardiovascular disease, n (%)	1 (16.7%)	8 (17.4%)	1 (25.0%)	0.266
BMI (kg/m^2^), n (%)				
18.5–23	1 (16.7%)	22 (47.8%)	2 (50.0%)	0.213
23–27.5	4 (66.7%)	22 (47.8%)	1 (25.0%)	
≥27.5	1 (16.7%)	2 (4.3%)	1 (25.0%)	
LVEF, n (%)				
<35%	3 (50.0%)	2 (4.3%)	1 (25.0%)	0.001
35–50%	2 (33.3%)	36 (78.3%)	2 (50.0%)	
>50%	1 (16.7%)	8 (17.4%)	1 (25.0%)	
TIMI score				
1–4	1 (16.7%)	29 (63.0%)	2 (50.0%)	0.010
4–8	5 (83.3%)	17 (37.0%)	2 (50.0%)	
Pre-TIMI flow				
TIMI-0	4 (66.8%)	14 (30.4%)	2 (50.0%)	<0.05
TIMI-1	2 (33.3%)	26 (56.5%)	2 (50.0%)	
TIMI-2	0	6 (13.0%)	0	
TIMI-3	0	0	0	
Post-TIMI flow				
TIMI-0	1 (16.7%)	0	0	<0.05
TIMI-1	0	2 (4.3%)	0	
TIMI-2	3 (50.0%)	8 (17.4%)	1 (25.0%)	
TIMI-3	2 (33.3%)	36 (78.3%)	3 (75.0%)	
Killip class				
Class-1	2 (33.3%)	30 (65.2%)	2 (50.0%)	<0.001
Class-2	1 (16.7%)	15 (32.6%)	1 (25.0%)	
Class-3	3 (50.0%)	1 (2.2%)	1 (25.0%)	
Total ischemic time (mins)				
<180	1 (16.7%)	5 (10.9%)	2 (50.0%)	0.006
180–360	1 (16.7%)	30 (65.2%)	1 (25.0%)	
>360	4 (66.7%)	11 (2.4%)	1 (25.0%)	
Number of vessels				
1	4 (66.7%)	16 (34.8%)	1 (25.0%)	0.293
2	1 (16.7%)	18 (39.1%)	2 (50.0%)	
3	1 (16.7%)	12 (26.1%)	1 (25.0%)	
Infarct location				
Anterior	5 (83.3%)	29 (63.0%)	1 (25.0%)	0.006
Non-anterior	1 (16.67)	17 (36.96)	3 (75.0)	

Complete resolution was seen in four patients (7.1%), partial resolution in 46 patients (82.1%) and no resolution in six patients (10.7%) on the ECGs recorded 90-120 min after pPCI (Table 3). Overall STE resolution median was 47.0% (39.6% and 57.9%). The extent of STE resolution ranged from 14.28% to 78.57%.

In multiple linear regression analysis, male sex (β coefficient 5.02; P=0.005), history of diabetes mellitus (β coefficient -4.40; P=0.006), hypertension (β coefficient 4.62; P=0.001), dyslipidemia (β coefficient 4.04; P=0.036), infarct location (β coefficient 6.77; P=0.002), TIMI score >4 (β coefficient 3.25; P<0.001), Killip class (β coefficient -11.97; P<0.001), total ischemic time >180 minutes (β coefficient 6.86; P<0.05), involvement of more than one vessel (β coefficient 2.35; P<0.05) and post-TIMI flow (β coefficient 7.56; P=0.003) were identified as the independent predictors of STE resolution (Table 4).

**Table 4:** Independent predictors of no STE resolution by multiple logistic regression analysis LVEF: left ventricular ejection fraction; STE: ST-segment elevation; TIMI: thrombolysis in myocardial infarction

Variable	β coefficient	95% confidence interval	P value
Sex	5.02	-1.42 to 8.71	0.005
Diabetes mellitus	-4.40	-10.73 to -1.33	0.006
Hypertension	4.62	-2.54 to 6.88	0.001
Dyslipidemia	4.04	-0.44 to 11.40	0.036
Infarct location	6.77	-6.43 to 13.82	0.002
TIMI score	3.25	0.75 to 4.15	<0.001
Killip Class	-11.97	-15.35 to -6.39	<0.001
LVEF	3.29	-0.52 to 1.48	0.05
Total ischemic time (mins)	6.86	-0.12 to -0.07	<0.001
Number of vessels involved	-2.97	-5.86 to 0.04	0.003
Post-TIMI flow	7.56	-0.52 to 0.30	0.003

Partial STE resolution was observed in 82.1% of patients in our study. An interesting observation of the study was that out of the six patients with no STE resolution, three patients (50%) achieved post-TIMI 2 flow and two patients (33.3%) achieved post-TIMI 3 flow (Table 3). The independent predictors of 30-day morbidity/mortality are given in Table 5.

**Table 5:** Independent predictors of 30-day morbidity/mortality by multiple logistic regression analysis LVEF: left ventricular ejection fraction; STE: ST-segment elevation; TIMI: thrombolysis in myocardial infarction

Variable	Odds ratio	95% confidence interval	P value
Sex	0.82	0.49–1.37	>0.05
Diabetes mellitus	1.27	0.74–2.19	>0.05
Location of infarct	3.37	1.24–9.15	>0.05
TIMI score	0.67	0.53–0.87	>0.05
Killip Class	0.47	0.36–0.56	>0.05
LVEF	0.02	0.01–0.18	0.001
STE resolution			
<30%	(Reference)		-
30-70%	0.05	0.01–0.33	0.02
≥70%	1.98	1.31–2.97	0.01
Total ischemic time (mins)	2.01	0.50–8.04	>0.05
No. of vessels involved	1.61	0.61–4.2	>0.05
Post-TIMI flow	0.09	0.02–0.44	0.03

**Figure 2:**
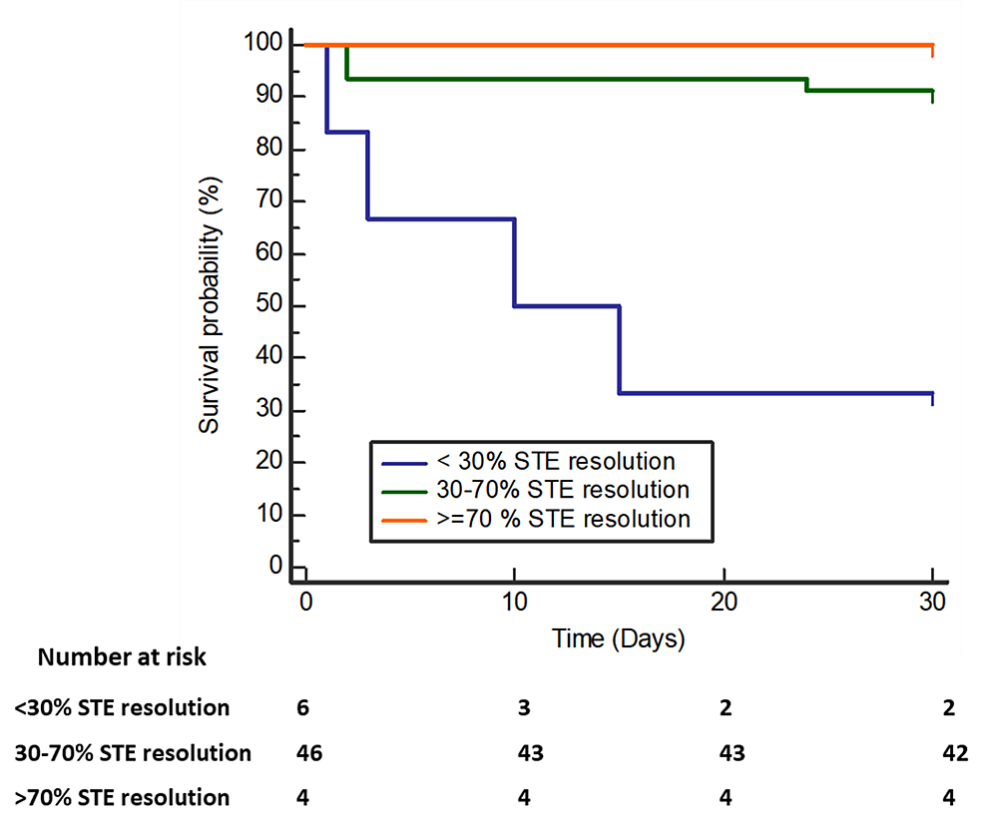
Kaplan-Meier curves for survival rate according to the extent of STE resolution STE: ST-segment elevation

In multivariable analysis, factors independently associated with 30-day mortality were extent of STE resolution [OR 0.05 (partial resolution) and 1.98 (complete resolution); 95% CI 0.01-0.33 (partial resolution) and 1.31-2.97 (complete resolution); P=0.02 (partial resolution) and P=0.01 (complete resolution)], LVEF (OR 0.02; 95% CI 0.01-0.18; P=0.001) and post-TIMI flow (OR 0.09; 95% CI 0.02-0.44; P=0.03). Kaplan-Meier survival analysis till 30 days revealed significant differences between the three groups (P<0.001) (Figure 2). At 30 days of follow-up, the primary endpoint of this study occurred in eight patients (Table 6).

**Table 6:** Clinical outcomes stratified by the extent of STE resolution STE: ST-segment elevation

Clinical outcome	Extent of STE resolution			Total (n=56)
	No Resolution (n=6)	Partial Resolution (n=46)	Complete Resolution (n=4)	
Cardiac event				
Death	1 (16.7%)	1 (2.2%)	0 (0.0%)	2 (3.6%)
Death due to heart failure	1 (16.7%)	0 (0.0%)	0 (0.0%)	1 (1.8%)
Heart failure	1 (16.7%)	0 (0.0%)	0 (0.0%)	1 (1.8%)
Recurrent ischemia	1 (16.7%)	1 (16.7%)	0 (0.0%)	2 (3.6%)
Re-infarction and recurrent ischemia	0 (0.0%)	1 (16.7%)	0 (0.0%)	1 (1.8%)
Stroke	0 (0.0%)	1 (16.7%)	0 (0.0%)	1 (1.8%)
Total	4 (66.4%)	4 (8.8%)	0 (0.0%)	8 (14.4%)
No cardiac event	2 (33.3%)	42 (91.3%)	4 (100.0%)	48 (85.6%)

The overall mortality was 5.36% (n=3). The mortality rate was 33.3% in patients with no STE resolution and 2.1% in patients with partial STE resolution. The comparison of survival curves by log-rank test with respect to prominent variables is given in Table 7. No mortality was observed in patients with complete STE resolution.

**Table 7:** Comparison of events observed versus events expected by log-rank test LVEF: left ventricular ejection fraction; STE: ST-segment elevation; TIMI: thrombolysis in myocardial infarction

Variable	Events observed	Events expected	P value
Diabetes mellitus			
Yes	7	3.38	0.009
No	1	4.62	
Infarct location			
Anterior	7	4.82	0.11
Non-anterior	1	3.18	
Killip class			
Class-1	2	5.08	0.001
Class-2	1	2.50	
Class-3	5	0.42	
Total ischemic time (mins)			
<180	0	0.61	0.56
180-360	5	5.39	
>360	3	2.00	
STE resolution			
1 (<30%)	4	0.66	<0.001
2 (<70–30%)	4	6.74	
3 (≥70%)	0	0.61	
LVEF			
<35%	5	0.58	0.001
35–50%	3	5.91	
>50%	0	1.52	
Number of vessels			
1	2	3.08	0.60
2	3	3.01	
3	3	1.91	
Post-TIMI flow			
0	1	0.02	0.001
1	2	0.14	
2	3	1.64	
3	2	6.20	

## Discussion

This is the first study exploring the utility of STE resolution in predicting 30-day mortality in Indian patients. We stratified patients according to the extent of STE resolution based on the ECGs recorded 90-120 minutes after initiation of reperfusion therapy. From a total of 56 patients, complete resolution (≥70%) was present in 7.1% of patients, partial resolution (<70-30%) in 82.1% of patients, and no resolution (<30%) in 10.7% of patients. Palmerini et al. in their similar study on a Northern Italian population found that 75% of patients presented with STE resolution (>50% STE resolution) and 22% (≤50% STE resolution) did not [19]. The noticeable difference in proportions is because of having a different number of sub-groups in the study. Having a single cut-off point, firstly, has the limitation of including a higher number of patients with still compromised LV function [20]. Secondly, the groups with lesser STE resolution comprise patients with a relatively favorable outcome. Following these observations of Schröder et al., we included two cut-off points, which created three groups of STE resolution [20].

Comparable with previous studies [20,21], 75% of patients with no STE resolution had a non-anterior infarction and 25% had an anterior infarction. The reverse was true in the case of patients with no STE resolution. Non-anterior infarct was present in 16.7% and anterior infarct was present in 83.3%. The main finding of our study was that patients who have complete STE resolution are less susceptible to a 30-day mortality risk as compared to patients who have partial or no STE resolution (P<0.05). Patients with no and partial STE resolution had a mortality rate of 33.3% and 2.1%, respectively. No mortality was seen in patients with complete STE resolution. MACE rates were lower in patients with incomplete STE resolution (8.8%) than in patients with no STE resolution (66.4%). The median STE resolution in our study was 47%, with 50% of the patients having an extent of STE resolution between 39.6% and 57.9%. Claeys et al. in their study on determinants of persistent STE observed 38% median STE resolution [22]. A study conducted by Schröder et al. showed that ST-segment resolution was the most powerful independent predictor of 35-day mortality [20].

Supporting our findings, Johanson et al. found that patients with a low-grade ST-segment resolution are at higher risk when experiencing an acute STEMI [23]. Continuous ST monitoring of patients with acute myocardial infarction yields important prognostic information after 60 min of observation and should be used for very early-risk stratification in these patients [23].

The baseline characteristics of the three groups differed significantly only in terms of hypertension. The clinical and procedural characteristics differed significantly in terms of LVEF, total ischemic time, post-procedural TIMI flow and infarct location. We observed a correlation between the extent of STE resolution, Killip class, and TIMI score. Stepwise regression analysis of baseline characteristics revealed that males and patients with comorbidities such as diabetes and hypertension were more likely to have no STE resolution following pPCI. Comorbidities have an association with impaired microvascular function after acute MI, resulting in a larger infarct size and poor functional recovery. Higher STE reflects a more extensive MI. This explains why patients in the current study with comorbidities and no STE resolution had higher STE than their counterparts before PCI.

Regardless of early recanalization of an occluded infarct artery, microcirculation reperfusion may still be hindered because of microvascular damage due to ischemia and/or reperfusion [22]. Persistent STE in an ECG performed shortly after angioplasty represents sustained cardiac transmural injury, impaired cardiac microcirculation and myocardial damage. Evident by the wide range of STE resolution in our study (14.28% to 78.57%), the extent of microvascular reperfusion injury was variable.

Multivariate logistic regression analysis of clinical and procedural characteristics found inability to achieve TIMI 3 flow, angioplasty in the left anterior descending artery territory, low LVEF, high TIMI score, higher Killip class, and longer total ischemic time as important predictors of not achieving complete STE resolution. Patients with persistent STE had longer ischemic time (>180 min). This observation was supported by van’t Hof et al. who found a strong relation between ischemic time and the extent of STE resolution. Lower ischemic time predicted a better STE resolution profile (P<0.005) [15].

Patients with a post-procedural TIMI flow 0 to 2 were at a higher risk of 30-day mortality than those with a post-procedural TIMI 3 flow. Post-intervention TIMI 3 flow was found in 73.21% of patients, indicating reperfusion. Similarly, Mehta et al. observed that compared with patients with TIMI flow grade 3, those with TIMI flow grades 0 to 2 were more likely to undergo coronary artery bypass graft surgery after PCI (20% vs. 5.4%). Unadjusted mortality was more than two-fold higher with TIMI flow grades 0 to 2 versus TIMI flow grade 3 (63% vs. 27%) [24]. Although post-TIMI 3 flow was achieved in 73.21% of patients, only 7.1% were able to achieve complete STE resolution. TIMI reflects epicardial rather than myocardial flow. However, the outcomes of PCI are better linked to myocardial flow which is reflected by STE resolution. This could explain the occurrence of mortality despite TIMI 3 flow restoration. STE resolution served as an independent predictor of 30-day mortality across all clinical variables, and even in patients with post-procedural TIMI 3 flow. Our results are in line with the findings of Palmerini et al. who found that STE resolution was an independent predictor of 30-day mortality in patients with TIMI 3 flow (P<0.0001) [19].

This study has a few limitations. First, this was a single-center study with a limited number of enrolled patients. Therefore, the results cannot be generalized to the overall Indian population presenting with STEMI. Second, we assessed STE resolution in a semi-quantitative way by comparing two 12-lead ECGs. This approach might be less accurate than continuous ST-segment tracking with automated analysis systems. Third, ST-segment analyses for this study were conducted by a single investigator, albeit blinded to patient and outcome data. Last, late ST-segment analysis (>90 min) may reduce the prognostic power of ST-segment resolution.

## Conclusions

Patients who have persistent STE after undergoing pPCI for STEMI have an increased risk of adverse outcomes, including mortality. The current study adds to this growing body of evidence, demonstrating that STE resolution also has prognostic utility in Indian patients undergoing pPCI for STEMI. Patients who exhibit persistent STE after pPCI are at increased risk of adverse clinical outcomes, including higher 30-day mortality. Therefore, close monitoring and additional therapeutic interventions should be considered for these patients. Identifying and treating persistent STE is an important aspect of STEMI management, and the use of STE resolution as a simple and affordable tool may help to improve outcomes in this patient population.
